# Supplementary figures and images for: The citrus tristeza virus p33 protein functions as a viroporin
Source: PLoS Pathog. 2025 Nov 24;21(11):e1013730. doi: 10.1371/journal.ppat.1013730 (PMC12671738; doi:10.1371/journal.ppat.1013730)

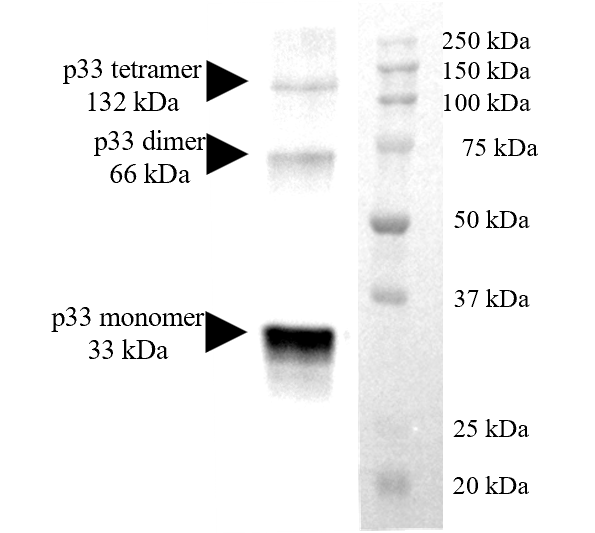

Supplement: S1 Fig — (TIF) [file ppat.1013730.s001.tif]
