# Supplementary material for: The citrus tristeza virus p33 protein functions as a viroporin
Source: PLoS Pathog. 2025 Nov 24;21(11):e1013730. doi: 10.1371/journal.ppat.1013730 (PMC12671738; doi:10.1371/journal.ppat.1013730)
Supplement: Attachment — Submitted filename: Response to Reviews.docx [file ppat.1013730.s003.docx]

**Response to reviewers’ comments**

**Part II – Major Issues: Key Experiments Required for Acceptance**

**Reviewer #1:** There are two previous reports about plant virus viroporins. Thus, the statement on lines 31-32, and equivalent everywhere in the manuscript, needs to be revised and remove the idea that this is the first report. This is despite the argument in lines 91-93. The conclusion does not change, this is not the first report of a viroporin in a plant virus.

**Response:** Thank you for the overall positive comments. To avoid any confusion, the statements were modified throughout the text (see lines 37-38, 110-114, 166-167, 807-809).

**Reviewer #2:** Some experimental details still need further validation. For instance, the reliability of the oligomeric state bands observed in SDS-PAGE under non-denaturing conditions, the composition of the solutions and blockers used in electrophysiological experiments, and the identification of vesicle-like structures and Hechtian strands observed under confocal microscopy. Also，it would be interesting to know how the vesicle structures formed by the p33 protein are correlated with its ion channel activity.

**Response:** Thank you for the overall positive comments. The comments raised here by Reviewer #2 are elaborated in detail in the “Minor Issues” section below, and each is addressed independently. Modifications were made where requested, and we answered the questions raised by the reviewer.

**Reviewer #3:** The conclusions that p33 is a viroporin will be substantially strengthened if mutations that affect the pore structure or ion channel activity can be confirmed. The effects of such mutations on oligomerization, ion channel, among other assays, should be tested. The same is true for the projected amphipathic helix. Key amino acids can be targeted to disrupt helix or amphipathicity to confirm such structure plays a key role in the formation of pore, oligomerization, ion channel, etc.

Fig. 4 A known viroporin should be included as a positive for comparison purposes: is p33 has strong or minimal activity compared to other well-characterized viroporins.

**Response:** Thank you for the constructive comments. We now added new experimental data comparing the wild type p33 and a mutant version of p33 containing a deletion of the 30 amino acids from the N-terminus of p33. Earlier, we demonstrated that this region of p33 is involved in the protein self-interaction (see ref. 32). Here, we show that the mutant had a totally different intracellular distribution, compared to the wild type p33, and was no longer localized on the plasma membrane (see lines 547-550 and a new Figure 7). Instead, the mutant formed irregular inclusion-like structures. Therefore, the mutant will not be expected to function as a channel-forming protein. As for the suggestion of mutating the amphipathic helix, our earlier study showed that a mutation of Tyrosine to Glycine in the amphipathic helix shifts GFP:p33’s localization from vesicles to amorphous cytoplasmic aggregates (see ref #22). We have added this information into the manuscript in the Results and Discussion sections (see lines 550-555 & 861-863). As it is no longer associated with vesicles and the plasma membrane, we do not expect the altered protein to have an ion chancel conductance either.

With agree with the other suggestions of the reviewer. However, regretfully, our collaborator who provided us with the materials and equipment for the electrophysiological assays (Dr. Papke) has retired, and neither equipment nor the frogs are available any longer. As such, further TEVC experiment cannot be performed in our lab.

**Part III – Minor Issues: Editorial and Data Presentation Modifications**

**Reviewer #1:**

1. The title needs to be re-structured, CTV is a plant virus.

**Response:** Thank you for the suggestion. The title was revised accordingly.

2. Current binomial names for virus species should be used.

**Response:** Thank you for the suggestion. The binomial species name were included at the first mention of every virus.

3. Figure legends are dispersed within the text: Lanes 232-237 are misplaced. Same for lanes 270-279, Fig 4 legend, and others.

**Response:** Thank you for the suggestion. This is the way the author guidelines specify the legends should be placed. However, at the request of the reviewer, the legends were deleted with tracked changes. If indeed the journal needs those in the text, the changes can be rejected and reverted. The legends are still placed with the figures at the end of the manuscript.

4. Tatineni et al., 2012 is not found in references.

**Response:**  Thank you for the suggestion. The above publication has been added to the references (ref. # 66).

5. Figure 5 legend needs to be revised for clarity.

**Response:** Thank you for the suggestion. The Figure 5 legend was revised under the image at the end of the document.

**Reviewer #2:**

Page 8, Line 157-158, Figure 1A, how to identify the cytoplasmic domain (CD) and external domain (ED) of the known viroporins and p33?

**Response:** Thank you for the suggestion. These domains have been already identified in earlier publications. This has been added to the text and the references cited (lines 196 – 197).

Page 8, Figure 1A, please specify which tools were used to predict the TMD.

**Response:** Thank you for the suggestion. The domain has been already identified in an earlier publication. This has been added to the text and the reference cited (lines 196 – 197).

Page 8, Line 183-185, Figure 1B, I noticed that the protein extraction process did not undergo sufficient denaturation (e.g., treatment with β-ME). If the protein structure is not fully unfolded, can its size be used as a reference in SDS-PAGE electrophoresis? Please provide native electrophoresis results or other evidence to support the conclusion that p33 is in a oligomeric state.

**Response:** Thank you for the comment. However, protein dimers or oligomers can also be observed on SDS-PAGE, which has been well documented in previous studies (a few examples: Pubmed ID: 11784321, 21917547, 25143246). Similar to those studies, we can observe oligomers of p33 in SDS-PAGE. So, we will stick to this method. The findings of the oligomers formation by p33 in this work is also in agreement with our previous demonstration of the ability of p33 to self-interact with the use of other techniques (see ref. 32).

Page 8, Figure 1C, please add a loading control to this figure. Besides, please specify the meaning of the numbers at the top of the figure in the figure legend.

**Response:** Thank you for the suggestion. A new Figure 1B with a loading control was included, and the old Figure 1B was provided as a supplementary figure (S1 Fig). Both the new 1B figure and the old one (S1 Fig) sufficiently support the formation of the oligomers by p33. The Figure 1C was removed as it does not add any further information. Additional clarification was added on lines 206-248.

Page 8, Figure 1, what is the difference in the protein extraction methods between Figure 1A and 1B? Please provide the details in the Methods section.

**Response:** Thank you for the suggestion. The difference between the purified p33 and crude extraction methods was added to methods section (lines 1018-1019).

Page 15, Line 315, LaCl3 is merely Ca2+ channel inhibitor, how can the influence of calcium-activated chloride channel be eliminated?

**Response:** Thank you for the comment. We have already provided a reference in which LaCl3 has been used (ref. 49). While the Ca-dependent chloride channels would not be affected, this is irrelevant to our work as we decided to avoid eliciting those endogenous currents altogether by not exceeding -150 mV as mentioned in the text on line 446. Furthermore, the effect of Ca2+-dependent chloride channels would also be observed in the control water-injected oocytes. But again, no blockers of any channels were necessary as we only lowered the voltage to -150 mV.

Page 15, Figure 4, according to other studies, the control group injected with water should show no current changes, why does the control group in your results also exhibit current changes？

**Response:** Thank you for the comment. Based on the published literature, when the membrane potential is lowered from +50 mV to -150 mV in oocytes, there should be at least a modest change observed. The extent of this change can vary between different batches of oocytes from different frogs. The approximately 200 nA change observed in our water-injected oocytes between +50 mV to -130 mV is not uncommon (see references below).

References (examples of modest current changes in water-injected oocytes):

1) Kottgen et al 2005 **FIGURE 4B** <https://www.embopress.org/doi/full/10.1038/sj.emboj.7600566>
2) Mannvile et al 2018 **FIGURE 3A**

<https://www.nature.com/articles/s41467-018-04266-w>

3) Mori et al 2018 **FIGURE 3A**

https://www.mdpi.com/2223-7747/7/3/61

The narrow scale we use in our graphs (a 1.9 µA scale in figure 4A) makes the change seem larger than for others who used a scale of typically 4 µA or taller on the vertical axis. The water trace looks rather flat when we use such scale as well (see figure below). In any case, the water-injected and p33-injected oocytes were from the same batch of oocytes, serving as what we believe is a confident control.

Page 15, Line 330, when conducting Na+ or K+ assays, retaining the presence of the other ion in the solution, even at a lower concentration, is not rigorous. Additionally, the influence of other ions, such as Ca2+ and Cl-, has not been excluded.

**Response:** Thank you for the comment. We followed the established protocols that previous viroporin reports have employed. These similarly contain low levels of calcium and salts such as KCl or NaCl, similar to our solutions (see references below). As such, we do not anticipate that the calcium or chloride levels present in our solutions have a significant influence on the results of our experiments.

References

1) Pinto et al. 1992

<https://www.sciencedirect.com/science/article/pii/009286749290452I>

2) Plugge et al. 2000

<https://www.science.org/doi/10.1126/science.287.5458.1641>

As for the Na and K assays and the presence of both in the solutions, we understand the reviewer’s concern. Our choices were instructed and motivated by previously published work. Given p33’s structural similarity to HIV-1 Vpu protein, we decided to use the same Na and K concentrations other researchers used in the Ringer’s solution for the Xenopus assays with Vpu (Schubert et al, 1996):

Source: https://febs.onlinelibrary.wiley.com/doi/abs/10.1016/S0014-5793%2896%2901146-5?sid=nlm%3Apubmed

This explains why the Ringer’s solution has a modest amount of KCl. Furthermore, given our results, we anticipate that the exclusion of Na or K from each assay would not influence our results. If p33 was significantly selective for K, then the increase of KCl from 2.5 mM to 50 mM should have caused a large increase in the currents in the high K bath, compared to those in the normal Ringer’s solution.

To explain why the NaCl was kept in the high K Ringer’s, our reasoning was to have a solution in which the sum of monovalent ions was similar. We also felt that substituting all of the NaCl for KCl, having a KCl of above 100 mM would cause too harsh a depolarization. Thus, we set the KCl at 50mM and kept the rest as NaCl. We also found a similar buffer to be employed by Chen et al. 2005: <https://www.sciencedirect.com/science/article/pii/S0006291X04027214#section.0010>

Page 15, Line 331, please provide the reference about “Most viroporins that conduct potassium also conduct sodium”, thank you.

**Response:** Thank you for the suggestion. The reference was added (ref. 4; line 462).

Page 16, Line 357-359, “It was postulated...to reach the” is an incomplete sentence.

**Response:** Thank you for the suggestion. The missing word “the plasmodesmata” was added to the sentence (line 479).

Page 17, Line 363, please write out the abbreviation on first use.

**Response:** Thank you for the suggestion. The abbreviation for VSR has already been introduced above in the text (line 138).

Page 17, Line 369, how to determine the vesicle structure? Are there corresponding markers? At the same time, a negative control is needed to exclude the role of p22 in vesicle formation.

**Response:** Thank you for the suggestion. In a previous study, we already showed that p33 forms vesicles that co-localize with the ER and Golgi markers, but not with a peroxisome marker (Bak and Folimonova, 2015; file:///C:/Users/User/Zotero/storage/EGPMTEE4/S0042682215003141.html). These vesicles were found only in the p33-expressing cells but not in the control cells expressing any VSR protein or mixtures of those (such as p19, HC-pro, and p24 VSRs).

As for p22, the TEM figures show the control treatment of an empty cassette + the p22 VSR-injected leaves did not exhibit any vesicles. So, we are confident that the formation of vesicles results from the expression of the wild type p33.

Page 18, Figure 6, why not use immunogold labeling to confirm the presence of p33 on the vesicles? Is the formation of vesicles dependent solely on the viral p33 protein?

**Response:** Thank you for the suggestion. We do not claim the formation of vesicles is solely dependent on p33, we only claim that p33 induces their formation, which is apparent by their absence in the control. Whether this is done independently by p33 or through the recruitment of other (host?) factors is an interesting future direction of our work and is yet to be studied.

We also added new data showing that the deletion of p33’s N-terminal 30 amino acids, which is involved in p33 self-interaction, abolishes its localization to vesicles (lines 547-550 and Figure 7). Furthermore, we also added our previous findings showing that the mutation of the amphipathic helix of p33 abolishes p33’s localization to the vesicles (lines 550-555). This suggests that only a functional self-interacting p33 localizes to these vesicles.

Page 19, Line 412-413, Figure 7, how to determine the Hechtian strands? Are there corresponding markers?

**Response:** Thank you for the comment. Hechtian strands are very characteristic structures that connect the plasma membrane to the cell wall known to appear during plasmolysis. There are no structures they could be mistaken for and markers are not necessary for their identification.

Page 29, Line639, the blocker LaCl3 mentioned earlier were not described in the methods section, please provide additional details.

**Response:** Thank you for the comment. The blocker was not used in our experiments. Only briefly mentioned as a method that some other use when they go beyond -150 mV, which we did not do.

**Reviewer #3:** The title starts with “Plant virus viroporins on the rise”, which gives a false impression that this is a review paper.

**Response:** Thank you for the suggestion. The phrase was deleted from the title.

L359, missing a word after “the”.

**Response:** Thank you for the suggestion. The word was added.
