# Supplementary material for: The citrus tristeza virus p33 protein functions as a viroporin
Source: PLoS Pathog. 2025 Nov 24;21(11):e1013730. doi: 10.1371/journal.ppat.1013730 (PMC12671738; doi:10.1371/journal.ppat.1013730)
Supplement: Attachment — Submitted filename: Response to Reviews 2_Oct 29.docx [file ppat.1013730.s004.docx]

**Response to reviewers’ comments**

**Reviewers' Comments:**

**Part I - Summary**

Reviewer #3: The authors have addressed most of my requests, though the retirement of a coauthor prevents them from performing TEVC assays to include a well-studied viroporin.

I have no further request.

**Response:** The authors thank the reviewer for the positive comments.

Reviewer #4: The manuscript by Aknadibossian et al. reports that the p33 of citrus tristeza virus (CTV) is a function viroporin. They first compared the structural similarities between p33 and known viroporins, then tested the viroporin activity using the electrode clamp assay, and finally investigated its subcellular localization and membrane remodelling ability. The discovery of p33 as the third plant virus-encoded viroporin is novel and interesting. Although the electrode clamp assay clearly showed the viroporin activity of p33, some experiments of this study are not consistent or are not related to the viroporin activity in my opinion (see below).

**Response:** Thank you for the overall positive comments. We have addressed the reviewer’s suggestions as indicated below.

**Part II – Major Issues: Key Experiments Required for Acceptance**

Reviewer #3: (No Response)

Reviewer #4: 1, the only experiment directly related to the ion channel activity is the electrode clamp assay. Although reliant, the use of only p33 wide-type but no p33 mutants was used is unacceptable (also noted by the reviewers in the previous review). The authors explained the availability of the equipment for the electrode clamp assay, which is acceptable. I think it is necessary to test the viroporin activity using the yeast complementation assay with the K+ uptake deficient yeast strain CY162 (commercially available).

**Response:** We made an extensive effort to follow the reviewer’s suggestion that involved the research activities over several months described below. We acquired the CY162 strain and engineered the constructs designed to express the wild type p33 (WT p33), a p33 lacking its transmembrane domain (p33ΔTMD), and three constructs for the expression of p33 mutants containing point mutations in the C-terminal TMD of p33 (S290C, L297W, S295C). We also built the constructs expressing tomato ACA10, which is a calcium ATPase transporter (Wang et al., 2011; DOI: 10.1105/tpc.111.088013), to be used as a negative control and Arabidopsis potassium channel protein 1 (KAT1) to serve as a positive control (Saito et al., 2017; DOI: 10.1080/19336950.2017.1372066). The PCR fragments were inserted into pENTR-D-TOPO vector (Thermo Fisher Scientific). The sequence-verified entry clones were recombinated with yeast expression pYL435 vector (Arabidopsis Biological Resource Center). The constructs were used in yeast complementation experiments. Yeast CY162 strain (Lifeasible) was transformed with the expression constructs following standard protocol. The transformed yeast cultures were grown on synthetic media with varying concentrations of potassium for 72 hr. As shown below in Figure 1A, the positive control potassium channel AtKAT1 allows the deficient strain to grow at 1 mM KCl, while the negative control could not complement the yeast at such low concentrations. With that, no meaningful results could be obtained with all the p33 variants as those could not be expressed in yeast: protein expression was not detected in the western blots with anti-p33 primary antibodies (Figure 1B). Western blotting was performed following the standard conditions developed for the detection of the p33 protein previously. Multiple attempts, which included trials with different yeast growth media, growth time, and variable concentrations of KCl, yielded the same results despite close attention to detail, constructs’ sequence verification, and repeats with additional versions of the p33 plasmid constructs.

Figure 1: Yeast CY162 complementation assay. (A) Growth of serially diluted cultures of transformed CY162 yeast. The p33 WT, p33dTMD, and p33 TMD point mutations (S290C, L297W, S295C) sequences were cloned in the yeast expression vector along with ACA10 as a negative control and AtKAT1 and a positive control. Liquid cultures were diluted to a final OD_600_ of 1.0 (SD1), then serially diluted 10-fold for SD2 and SD3. 10uL of each diluted culture was used for each spot on the synthetic growth media with 50, 5, or 1 mM KCl. The pictures were taken after 72 hours of incubation at 30°C. (B) Western blot of the transformed yeast. The samples were collected from single colony-inoculated yeast cultures after a 48-hr growth period. Samples were resuspended in buffer containing 8M urea and 5% beta-mercaptoethanol and heated at 100°C for 10 minutes. α-p33 primary antibodies (1:500) and α-rabbit secondary antibodies (1:5000) were used for western blotting. The protein extract from the *N. benthamiana* leaves expressing the p33 protein was loaded as a positive control.

The p33 protein has a history of being difficult to express in heterologous expression systems, which is possibly exacerbated by its high hydrophobicity and its cysteine-rich nature. While we successfully express WT p33 in *N. benthamiana*, we have failed to express p33 in:

1) Baculovirus expression system (unpublished results).
2) *E. coli* system (Kang et al., 2021; DOI: 10.3390/v13112129). After our initial failure to express a histidine-tagged WT p33 in *E. coli,* we attempted to express it fused to a GST tag, which is known to enhance protein solubility.
3) Even in *N. benthamiana,* small modifications to the protein N-terminal domain also altered the protein localization from the cellular membranes to cytoplasmic aggregates, as demonstrated by GFP:p33ΔN30 deletion mutant in Figure 7 in the manuscript.

The above examples illustrate that, unfortunately, it is not surprising that we were not able to express p33 in yeast. On the other hand, our success in Xenopus oocytes is also not surprising as this system has shown excellent capacity to express a wide range of membrane proteins and ion channels originating from the different kingdoms of life, including plants (Theodoulou & Miller, 1995; DOI: 10.1007/BF02789106; Miller & Zhou, 2000; DOI: 10.1016/s0005-2736(00)00148-6; Limon & Mattei, 2023; DOI: [10.3390/membranes13100831](https://doi.org/10.3390/membranes13100831)).

In conclusion, despite investing several months of effort, we were unable unfortunately to perform this assay due to the incompatibility of p33 with this yeast expression system.

2, The results in Fig. 3A and B are not related to the putative a-helix forming the channel, which is located at the C-terminus and should therefore be deleted.

**Response:** Thank you for your comment. The results presented in Fig. 3 demonstrate the parallel between the p33 protein organization and that of known viroporins and, thus, it is relevant in support of the viroporin function of p33. We think this comparison will also benefit the broader reader audience. So, the figure was kept in the manuscript.

3, The relationship between viroporin activity and phosphorylation is too far-fetched unless the authors have shown that p33 mutations with either a D or A substitution of S8 have increasesed or decreased viroporin activity. Therefore, Fig. 3C can also be deleted.

**Response:** Thank you for your comment. This information is relevant to the viroporin function of p33 and may help future studies on the viroporin activity of p33. So, the figure was kept in the manuscript.

4, Fig. 5 to 7 are also not strongly related to the viroporin activity; however, analysis of the localization of p33 during CTV infection may shed light on the function of p33 viroporin during viral infection.

**Response:** Thank you for your positive comment regarding the data presented in these figures. We agree with the reviewer’s assessment.

**Part III – Minor Issues: Editorial and Data Presentation Modifications**
Please use this section for editorial suggestions as well as relatively minor modifications of existing data that would enhance clarity.

Reviewer #3: (No Response)

Reviewer #4: 1, please note that the arrangement of CD and ED in p33 is opposite to that of IAV M2, HIV Vpu and SAR Cov E in Fig. 1A.

**Response:** Thank you for the comment. We are aware of the difference in orientation, which is the reason why we have labelled the CD and ED for each individual sequence to avoid confusion. The alternative would be to flip the sequence to align the CD and ED tags, which would result in the amino acid numbering starting at the opposite end for p33. To make it easier for the readers, we prefer to keep the conventional NH2🡺COOH amino acid sequence instead of flipping it.

2, lines 179-183, why does the addition of SGS and 8M urea fail to disrupt p33 tetramers? Is it possible that disulfide bonds were involved in the formation of p33 tetramers? This can be confirmed by the addition of DTT.

**Response:** Thank you for the comment. Our buffer indeed contained 100 mM DTT. The failure of DTT to disrupt these bonds actually indicates that it is independent of disulfide bonds. The curiosity is warranted as these are very harsh conditions for oligomers to persist. The oligomerization of these proteins is a complex topic meriting a study of their own, but existing as an oligomer under the denaturing conditions we have reported is indeed possible and has previously been reported for ligand-gated ion channels such as the nicotinic α7 and 5-hydroxytryptamine 3A (5HT3A) receptors (Nicke et al., 2004; DOI: [10.1016/j.febslet.2004.08.035](https://doi.org/10.1016/j.febslet.2004.08.035)). In that study, both receptors show multiple bands of oligomers in the presence of SDS, 8M Urea, and 100 mM DTT, similar to p33. This information has been added to the manuscript (lines 183-187).

3, line 207, please delete the context in parentheses as it has already been described in line 189.

**Response:** Thank you for your comment. The text has been deleted.

4, the scale bars in Fig. 5-7 are of different colors, thicknesses, location, and sizes, so keep consistent.

**Response:** Thank you for your comment. To improve consistency, we have:

- Changed the style of scale bars in Figure 7 to the same as the other figures.
- Changed the color of the scale bars in Figure 8 to white, similar to all other figures.

5, Please check the scale bars between panels A and B in Fig. 5.

**Response:** Thank you for your comment. We have double checked the scale bars and did not find any issue with them. The size of the vesicles (as determined by the scale bar) is consistent with previous observations in our lab (Bak and Folimonova, 2015; DOI: [10.1016/j.virol.2015.07.005](https://doi.org/10.1016/j.virol.2015.07.005). Figure 5B is not a magnification of Figure 5A, but rather a distinct image taken at a separate timepoint at which larger vesicles and MVBs can be observed, as is stated in the figure legend and the text.
